# Fistule carotido-caverneuse indirecte post traumatique: diagnostic indirect en contexte limitée

**DOI:** 10.11604/pamj.2016.24.72.9488

**Published:** 2016-05-17

**Authors:** Yannick Bilong, Chantal Nanfack Ngoune

**Affiliations:** 1 Département d'Ophtalmologie, Faculté de Médecine et des Sciences Biomédicales de l'Université de Yaoundé I, Cameroun; 2 Hôpital Gynéco-Obstétrique et Pédiatrique de Yaoundé, Cameroun

**Keywords:** Fistule carotido-caverneuse, exophtalmie, Cameroun, Carotid cavernous fistula, exophtalmos, Cameroon

## Image en médecine

La fistule carotido-caverneuse indirecte est une communication anormale entre de multiples artérioles provenant des branches durales ou méningées de l'artère carotide externe et/ou de l'artère carotide interne et le sinus caverneux. Son diagnostic de certitude a recours à l'artériographie carotidienne, mettant en évidence la fistule. Or, cet examen n'est pas disponible dans notre contexte d'exercice, d'où l'intérêt des signes indirects cliniques (exophtalmie avec dilatation des vaisseaux conjonctivaux en tête de méduse) et de l'angio scanner cérébral (dilatation de la veine ophtalmique supérieure) pour évoquer le diagnostic et éliminer les différentiels (tumeur orbitaire, exophtalmie thyroïdienne, pseudo tumeur inflammatoire, etc.). Nous rapportons le cas, d'un patient âgé de 58 ans venu consulter pour exophtalmie et baisse d'acuité visuelle gauche progressive évoluant depuis un an. Son histoire rapportait un traumatisme orbitaire gauche lors d'un accident de la voie publique trois ans plus tôt. L'exophtalmie gauche était axile, réductible, non pulsatile, sans souffle audible, indolore, sans limitation des mouvements oculaires, ni adénopathie satellite (A). L’œil gauche avait une acuité visuelle de 1/10 P10, une hypertonie à 28 mmHg, les vaisseaux conjonctivaux et rétiniens étaient dilates (A, B). L'examen de l’œil droit et le bilan thyroïdien étaient normaux. L'angio-scanner cérébral montrait une dilatation de la veine ophtalmique supérieure gauche (C). L'insuffisance du plateau technique, sur le plan endovasculaire donnant recours à une embolisation, nous a obligés à faire une prise en charge symptomatologique: larme artificielle et collyre hypotenseur.

**Figure 1:**
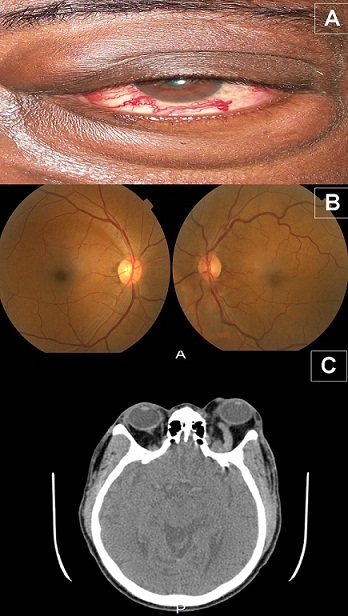
Signes indirect d'une fistule carotido-caverneuse indirecte. A) exophtalmie avec vaisseaux conjonctivaux dilatés en tète de méduse et chémosis palpébral; B) rétinographie: dilatation et tortuosité des veines rétiniennes dans l’œil gauche; C) Angio-scanner: dilatation de la veine ophtalmique supérieure gauche

